# Sequence-based GWAS and post-GWAS analyses reveal a key role of *SLC37A1*, *ANKH*, and regulatory regions on bovine milk mineral content

**DOI:** 10.1038/s41598-021-87078-1

**Published:** 2021-04-06

**Authors:** Marie-Pierre Sanchez, Dominique Rocha, Mathieu Charles, Mekki Boussaha, Chris Hozé, Mickaël Brochard, Agnès Delacroix-Buchet, Philippe Grosperrin, Didier Boichard

**Affiliations:** 1 Université Paris-Saclay, INRAE, AgroParisTech, GABI, 78350 Jouy-en-Josas, France; 2 Allice, 75012 Paris, France; 3 Umotest, 01250 Ceyzériat, France; 4 Conseil Elevage 25-90, 25640 Roulans, France

**Keywords:** Animal breeding, Gene regulation, Genetic association study

## Abstract

The mineral composition of bovine milk plays an important role in determining its nutritional and cheese-making value. Concentrations of the main minerals predicted from mid-infrared spectra produced during milk recording, combined with cow genotypes, provide a unique opportunity to decipher the genetic determinism of these traits. The present study included 1 million test-day predictions of Ca, Mg, P, K, Na, and citrate content from 126,876 Montbéliarde cows, of which 19,586 had genotype data available. All investigated traits were highly heritable (0.50–0.58), with the exception of Na (0.32). A sequence-based genome-wide association study (GWAS) detected 50 QTL (18 affecting two to five traits) and positional candidate genes and variants, mostly located in non-coding sequences. In silico post-GWAS analyses highlighted 877 variants that could be regulatory SNPs altering transcription factor (TF) binding sites or located in non-coding RNA (mainly lncRNA). Furthermore, we found 47 positional candidate genes and 45 TFs highly expressed in mammary gland compared to 90 other bovine tissues. Among the mammary-specific genes, *SLC37A1* and *ANKH*, encoding proteins involved in ion transport were located in the most significant QTL. This study therefore highlights a comprehensive set of functional candidate genes and variants that affect milk mineral content.

## Introduction

Bovine milk contains many essential nutrients, such as lactose (~ 48 g/L), fatty acids (~ 37 g/L), proteins (~ 34 g/L), and minerals (~ 9 g/L). Although less abundant than other solid components of milk, the major minerals—potassium (K), calcium (Ca), phosphorus (P), sodium (Na), and magnesium (Mg)—have an important effect on both human health and the cheese-making process. In humans, all of these minerals are necessary for many vital functions and therefore for the maintenance of good health. Dairy products can represent an important source of minerals in the human diet, especially of well-assimilated Ca^1^. In milk, minerals are found either in solution (soluble fraction) or in colloidal form (insoluble fraction). Some minerals are exclusively found in the soluble fraction (e.g., K and Na) while others exist in both fractions (e.g., Ca, P, and Mg). In the soluble fraction, Ca, P, and Mg exist in different forms, including ions and salts (phosphates and citrates), while in colloidal form, they are associated with casein molecules in the micelles and play a role in the structure and stability of these assemblages during the cheesemaking process^2^. Higher mineral concentrations are therefore associated with improved coagulation properties of milk^3,4^ and could enhance the nutritional value for human consumers.

Despite the potential benefits to human nutrition and milk processing, little is known about the genetic factors that influence milk mineral composition, mainly because the determination of mineral content via reference analyses is costly and time-consuming. A number of studies have reported genetic variation in milk mineral composition^5–11^, but to our knowledge, only two studies have conducted genome-wide association studies (GWAS) to investigate the genomic regions associated with these traits^9,12^. Both of those prior studies used 777 k SNPs and a relatively small sample of cows. As an alternative to reference analyses, mid-infrared (MIR) spectrometry can predict various milk components, including mineral fractions^13–15^, quickly and cheaply. Because of these advantages, milk MIR spectra are routinely recorded and stored. The combination of this technology with i) the widespread genotyping of cows for genomic selection, ii) the availability of whole-genome sequence (WGS) data from the 1000 Bull Genomes Project^16^, and iii) ever-increasing knowledge of the bovine genome^17,18^ creates the possibility of large-scale, high-resolution analyses for identification of the genes and variants that affect the mineral content of milk. We have previously applied this approach—whole-genome sequence-based GWAS combined with MIR predictions—to investigate milk protein composition^19^ and cheese-making traits^20^, and in both cases we succeeded in highlighting functional candidate genes. In particular, the genes *SLC37A1* and *ANKH* were strongly linked with milk quality; both encode transmembrane proteins involved in ion transport and are therefore likely to have an effect on milk mineral composition. However, in these genes, as well as in other genes we identified, the variants with the most significant effects were mostly found in non-coding regions with limited annotation, which made it difficult to distinguish the causal variant. This pattern is quite general and many studies have reported the major role of regulatory variants in the architecture of complex traits^21^. To address this challenge, further investigation of non-coding regions is needed, particularly with respect to binding sites of transcription factors and non-coding RNA which could regulate gene expression.

The main objective of this study was to identify the best candidate genes and variants that might affect the content of Ca, P, Mg, K, Na, and citrate in milk, as predicted from MIR spectra. For this, we first conducted a GWAS on imputed WGS data of 19,586 Montbéliarde cows, and then performed post-GWAS analyses using different sources of annotation data to further refine our results.

## Results

We analyzed six traits predicted from MIR spectra in Montbéliarde cows, representing the mineral (Ca, P, Mg, K, and Na) and citrate content of milk. MIR prediction equations originated from the Optimir project^14,15^. The accuracies of these MIR predictions, as assessed by the coefficient of determination (R^2^) in a validation population (Table 1), ranged from 0.68 to 0.90, with the exception of Na (0.44).

**Table 1:** Mean, standard deviation (SD), and accuracy (R^2^), estimated by cross-validation, of mid-infrared (MIR) predictions for concentrations of minerals and citrate in milk from Montbéliarde cows.

Trait	Abbrev	Mean	SD	R^2^ MIR prediction
Calcium, in mg kg^−1^ of milk	Ca	1161.4	92.6	0.82
Phosphorus, in mg kg^−1^ of milk	P	1007.0	77.5	0.75
Magnesium, in mg kg^−1^ of milk	Mg	1473.8	104.5	0.77
Potassium, in mg kg^−1^ of milk	K	100.5	7.2	0.68
Sodium, in mg kg^−1^ of milk	Na	341.7	44.5	0.44
Citrate, in g kg^−1^ of milk	Citrate	8.27	1.49	0.90

### Heritability and genetic correlation estimates for mineral and citrate content

Genetic parameters, i.e. heritabilities (h^2^) and genetic correlations (r_g_), were estimated for milk mineral and citrate content, as predicted from more than 1 million test-day records from 126,873 cows (Table 2). At the test-day level, heritability estimates were moderate for Na content (h^2^ = 0.32) but higher for other minerals (h^2^ = 0.50 to 0.56) and citrate (h^2^ = 0.48). Na was negatively and poorly correlated with other minerals (-0.23 ≤ r_g_ ≤  − 0.02) and citrate (r_g_ =  − 0.15), while the levels of most other minerals were generally positively correlated (0.11 ≤ r_g_ ≤ 0.60); the exception was the relationship between Ca and K (r_g_ =  − 0.22). Values of the genetic correlation between citrate and mineral levels in milk ranged quite broadly, depending on the mineral: null with K (r_g_ = 0.01 with K), slightly negative with Na (r_g_ =  − 0.15) and P (r_g_ =  − 0.16), and highly positive with Ca (r_g_ = 0.57) and Mg (r_g_ = 0.59).

**Table 2:** Estimates of heritability (in bold, diagonal) and genetic correlation (above the diagonal) for the concentrations of minerals and citrate in milk (SE < 0.01).

	Ca	P	Mg	K	Na	Citrate
Ca	**0.50**	0.34	0.60	− 0.22	− 0.20	0.57
P		**0.56**	0.58	0.39	− 0.23	− 0.16
Mg			**0.52**	0.11	− 0.02	0.59
K				**0.53**	− 0.05	0.01
Na					**0.32**	− 0.15
Citrate						**0.48**

### QTL identified in GWAS

**Figure 1:**
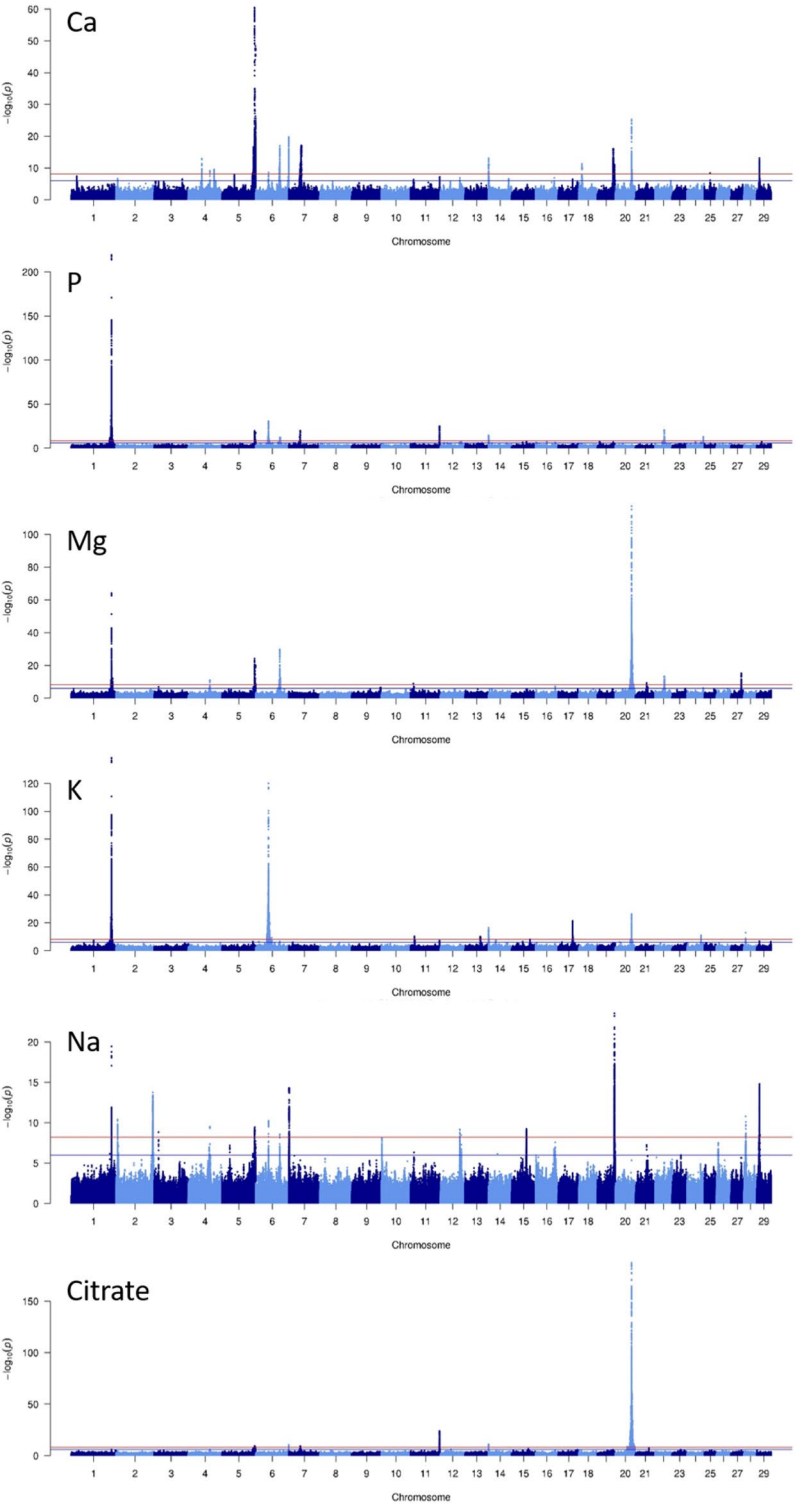
–log_10_(*P*) value of the effect of variants on milk mineral (Ca, P, Mg, K, and Na) or citrate content plotted against their position on *Bos taurus* autosomes.

**Figure 2:**
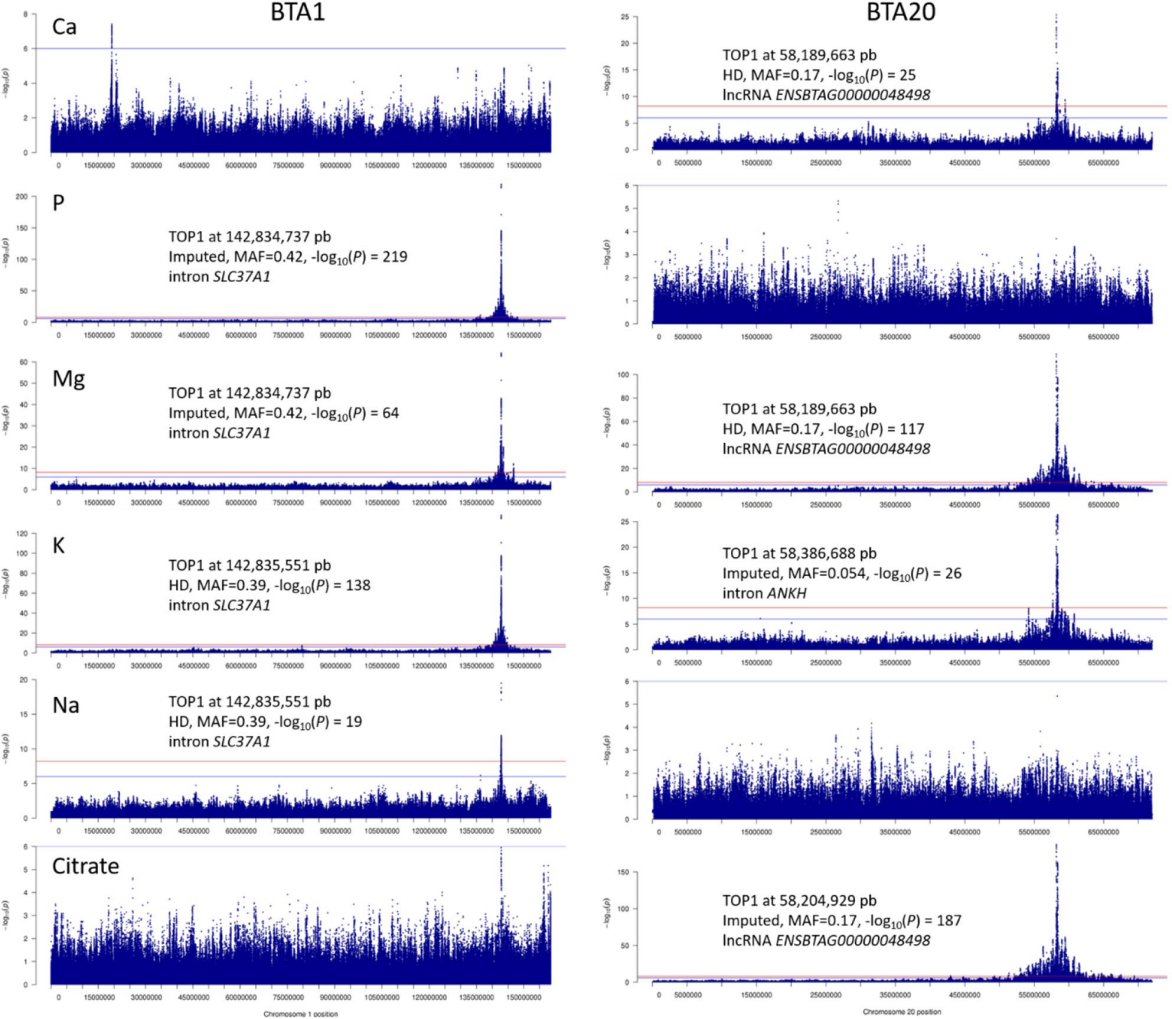
–log_10_(*P*) values of the effects of variants on milk mineral (Ca, P, Mg, K, and Na) or citrate content plotted against their position on *Bos taurus* autosomes 1 and 20.

We conducted single-trait GWASs on imputed WGSs from 19,586 cows (12,907,802 variants) and primarily identified 96 trait x region combinations with significant effects (-log_10_(P) ≥ 8.4) on milk mineral and citrate composition by applying the procedure described in the Methods section (Fig. 1). In two genomic regions (a 12Mbp-region on BTA1 and a 27Mbp-region on BTA20), the single marker analyses identified multiple trait x region combinations (10 and 15, respectively). Therefore, conditional analyses including the most significant variant identified in each region was applied to decipher if significant variants were due to linkage disequilibrium (LD) with the same causal mutation or to the presence of multiple causal mutations. These analyses led to the exclusion of 4 and 9 trait x region combinations on BTA1 and BTA20, respectively, resulting in 83 independent QTL. These QTL corresponded to 50 genomic regions located on *Bos taurus* (BTA) autosomes 1, 2, 3, 4, 5, 6, and 7 (Table 3) and 11, 12, 13, 14, 15, 17, 18, 19, 20, 21, 22, 24, 25, 27, 28, and 29 (Table 4). Of these, 18 regions had effects on several traits (2 to 5), while 32 affected only one trait. The four regions with the most significant effects (-log_10_(P) ≥ 50) each affected four to five traits; these were located on BTA 1 (~ 142.8 Mbp, for P, K, Mg, and Na; Fig. 2), 20 (~ 58.2 Mbp, for citrate, Mg, K, and Ca; Fig. 2), 6 (~ 45.3 Mbp, for K, Ca, P, and Na), and 5 (~ 116.4 Mbp, for Ca, Mg, P, citrate, and Na). Ten other regions with smaller but still highly significant effects (20 ≤  − log_10_(P) < 50) were located on BTA 5 (~ 119.0 Mbp), 6 (~ 43.3, 48.1, and 85.6 Mbp), 11 (~ 103.2 Mbp), 17 (~ 50.8 Mbp), 19 (~ 60.6 Mbp), 20 (~ 55.9 and 60.8 Mbp), and 22 (~ 32.8 Mbp); these also generally affected multiple milk components. All remaining significant QTL regions (8.4 ≤  − log_10_(P) < 20) were spread across 22 different autosomes. Overall, 6 to 21 different QTL were identified for each trait, and their cumulative effects explained from 19.8% to 45.5% of the genetic variance of a given trait (Table 5). The most significant QTL, located on BTA1 and BTA20, explained more than 23% of the genetic variance of P and citrate, respectively.

**Table 3:** QTL identified for milk mineral and citrate content on *Bos taurus* (BTA) autosomes 1 to 10 (when a QTL region affected multiple traits, the most significantly affected trait is indicated in bold).

Region	QTL	Trait	BTA	Confidence Interval of the QTL				Variant with the most significant effect (frequency *p* and effect *b* of the ALT allele)								
				From (bp)	To (bp)	# variants	# genes	Position (bp)	ID	*p*	R^2^	−log_10_P	*b*	SE	Name of gene(s)	Impact
1	1	P	1	135,384,680	136,262,601	8	3	136,262,601	rs210630730	0.199	0.69	11.5	8.57	1.23	TMEM108	Intron
2	4	Mg	1	142,826,156	142,837,365	9	1	142,834,737	rs109459130	0.576	0.85	64.1	1.48	0.09	SLC37A1	Intron
**2**	**5**	**P**	**1**	**142,826,156**	**142,835,551**	**7**	**1**	**142,834,737**	**rs109459130**	**0.576**	**0.85**	**219.3**	**30.2**	**0.95**	**SLC37A1**	**Intron**
2	6	K	1	142,826,156	142,838,477	31	1	142,835,551	rs109717634	0.613	1.00	138.1	22.4	0.89	SLC37A1	Intron
2	7	Na	1	142,826,156	142,835,551	7	1	142,835,551	rs109717634	0.613	1.00	19.5	− 3.77	0.41	SLC37A1	Intron
3	9	Mg	1	145,114,056	146,804,023	64	3	146,775,338	rs43048006	0.630	0.33	10.3	− 0.70	0.11	–	–
4	11	Na	2	5,738,340	7,483,560	104	7	5,739,439	rs209271012	0.111	0.86	10.4	3.85	0.58	LOC104971101	lncRNA
5	12	Na	2	131,111,763	131,742,987	206	9	131,197,793	rs133533873	0.672	0.99	13.8	2.84	0.37	ALPL	Intron
6	13	Na	3	15,459,107	15,540,579	7	6	15,470,670	rs110073735	0.026	0.92	8.9	− 5.20	0.86	EFNA1 / LOC107132270	Intron / lncRNA
7	14	Ca	4	48,711,128	48,835,496	22	4	48,711,294	rs381111559	0.291	0.59	13.0	− 10.5	1.42	SLC26A4	Intron
8	15	Na	4	75,153,659	77,188,370	14	7	77,147,308	rs386097640	0.024	0.87	9.5	6.65	1.06	GCK	Intron
8	16	Ca	4	77,147,308	77,257,775	9	7	77,173,366	rs133824313	0.025	0.98	9.1	14.8	2.40	GCK	Intron
**8**	**17**	**Mg**	**4**	**77,037,103**	**77,188,370**	**8**	**4**	**77,173,366**	**rs133824313**	**0.025**	**0.98**	**11.1**	**1.34**	**0.20**	**GCK**	**Intron**
9	18	Ca	4	91,807,789	92,192,288	182	6	92,192,288	rs42451422	0.028	0.95	9.5	13.4	2.13	LRRC4 / SND1	Intron / Intron
10	19	Ca	5	108,256,181	109,217,271	87	11	109,141,258	rs383141568	0.570	0.82	8.4	5.73	0.98	BID	Intron
11	20	Ca	5	111,637,039	111,848,859	47	7	111,745,513	rs384814366	0.231	0.94	16.6	− 9.38	1.11	MRTFA = MKL1	Intron
**12**	**21**	**Ca**	**5**	**116,403,262**	**116,463,104**	**48**	**8**	**116,429,840**	**rs382268272**	**0.049**	**0.58**	**60.5**	**− 34.8**	**2.11**	**LOC101903383**	**lncRNA**
12	22	Na	5	116,403,262	116,907,862	72	17	116,429,922	rs110716509	0.050	0.67	9.3	5.05	0.81	LOC101903383	lncRNA
12	23	Citrate	5	116,403,262	116,468,630	50	8	116,439,760	rs110048000	0.042	0.56	9.5	− 0.25	0.04	PPARA	Intron
12	24	Mg	5	116,403,806	116,463,104	36	6	116,448,403	rs110659119	0.048	0.97	24.2	− 1.38	0.13	PPARA	Intron
12	25	P	5	116,420,172	117,323,874	28	5	116,448,403	rs110659119	0.048	0.97	19.5	− 13.5	1.46	PPARA	Intron
**13**	**26**	**Ca**	**5**	**119,066,644**	**119,069,138**	**25**	**1**	**119,068,337**	**rs134583661**	**0.046**	**0.95**	**48.4**	**− 24.8**	**1.69**	**–**	**Intergenic**
13	27	Mg	5	119,066,644	119,069,138	25	1	119,068,337	rs134583661	0.046	0.95	20.1	− 1.29	0.14	–	Intergenic
13	28	P	5	119,066,644	119,069,138	25	1	119,068,337	rs134583661	0.046	0.95	17.9	− 13.2	1.50	–	Intergenic
14	29	K	6	40,012,262	40,518,049	18	2	40,024,540	rs43610452	0.064	0.99	11.7	− 11.6	1.66	SLIT2	Intron
15	30	P	6	42,855,236	43,032,274	17	3	42,870,371	rs42610629	0.523	0.97	9.2	5.59	0.91	–	Intergenic
16	31	K	6	43,297,568	43,322,676	12	1	43,322,676	rs43463132	0.778	0.97	40.7	13.2	0.98	–	Intergenic
17	32	P	6	45,018,531	45,401,570	78	4	45,056,055	rs378008740	0.713	0.76	30.6	12.7	1.09	–	Intergenic
**17**	**33**	**K**	**6**	**45,018,882**	**45,401,570**	**66**	**4**	**45,328,581**	**rs135848932**	**0.174**	**1.00**	**120.0**	**− 27.4**	**1.17**	**SEL1L3**	**Intron**
17	34	Ca	6	45,328,392	45,411,116	17	4	45,393,335	rs134399380	0.183	0.91	8.6	7.24	1.22	–	Intergenic
17	35	Na	6	45,215,035	45,401,570	17	4	45,401,485	rs108972810	0.177	0.99	10.2	− 3.39	0.52	SMIM20 / LOC112447060	Upstream BS DOF5.3 / lncRNA
18	36	K	6	47,687,737	49,094,600	79	1	48,145,964	rs43461552	0.336	0.92	26.9	− 10.6	0.98	–	Intergenic
19	37	K	6	50,277,420	51,315,936	82	2	51,311,430	rs110769289	0.555	0.53	9.1	− 5.72	0.93	–	Intergenic
20	38	K	6	56,960,135	56,970,541	6	1	56,968,022	rs134533189	0.091	0.41	9.4	− 8.37	1.34	RELL1	Intron
21	39	Na	6	85,409,619	86,104,900	312	15	85,512,651	rs110772556	0.660	0.98	8.6	2.79	0.47	–	Intergenic
**21**	**40**	**Mg**	**6**	**85,587,260**	**85,599,456**	**41**	**2**	**85,594,649**	**rs209134503**	**0.481**	**0.85**	**29.8**	**− 1.09**	**0.10**	**ODAM**	**Synonymous**
21	41	Ca	6	85,406,891	85,599,456	44	5	85,594,717	rs135113545	0.485	0.79	17.0	− 10.6	1.24	ODAM	Intron
21	42	P	6	85,564,407	87,276,896	1004	6	87,033,142	rs110121930	0.613	1.00	12.3	− 7.88	1.09	–	Intergenic
22	43	Mg	6	88,380,434	88,436,951	4	1	88,390,406	rs133413027	0.252	0.99	9.8	− 0.56	0.09	–	Intergenic
23	44	Citrate	6	117,331,679	117,456,476	30	7	117,407,851	rs43489995	0.306	0.30	10.4	0.22	0.03	TMEM175 / DGKQ	Downstream / Synonymous
**23**	**45**	**Ca**	**6**	**117,331,679**	**117,457,454**	**42**	**6**	**117,442,167**	**–**	**0.407**	**0.41**	**19.8**	**12.7**	**1.37**	**GAK**	**Intron**
24	46	Na	7	1,055,458	1,365,056	387	3	1,057,563	rs210648426	0.346	0.91	14.3	− 2.85	0.36	RASGEF1C	Intron
25	47	Ca	7	37,353,946	39,223,460	24	16	39,194,875	rs41567828	0.068	0.99	9.1	12.8	2.08	LOC107132625	lncRNA
**25**	**48**	**P**	**7**	**38,917,914**	**40,541,631**	**10**	**5**	**40,541,609**	**rs1116258782**	**0.009**	**0.22**	**16.9**	**31.7**	**3.71**	**–**	**Intergenic**
25	49	Ca	7	39,528,343	42,832,365	179	16	41,321,781	rs209051255	0.059	0.76	11.6	18.4	2.63	ENSBTAG00000053872	Upstream BS FOXD3
26	50	Ca	7	43,090,012	44,848,268	250	19	44,836,642	rs210107035	0.068	0.98	17.1	21.6	2.51	FSTL4 / LOC101902643	Intron / lncRNA

**Table 4:** QTL identified for milk mineral and citrate content on *Bos taurus* (BTA) autosomes 11 to 29 (when a QTL affected multiple traits, the most significantly affected trait is indicated in bold).

Region	QTL	Trait	BTA	Confidence Interval of the QTL				Variant with the most significant effect (frequency *p* and effect *b* of the ALT allele)								
				From (bp)	To (bp)	# variants	# genes	Position (bp)	ID	*p*	R^2^	−log_10_P	*b*	SE	Name of gene(s)	Impact
27	51	K	11	13,798,363	15,368,263	563	7	14,405,285	rs110177775	0.102	0.96	10.3	8.04	1.22	–	Intergenic
28	52	Citrate	11	103,239,082	103,277,147	279	5	103,244,306	rs435710868	0.514	0.85	23.9	0.21	0.02	ENSBTAG00000048091	Downstream
**28**	**53**	**P**	**11**	**103,239,082**	**103,277,147**	**278**	**6**	**103,250,796**	**rs379692402**	**0.473**	**0.72**	**25.0**	**− 11.8**	**1.13**	**–**	**Intergenic**
29	54	Na	12	68,052,340	69,715,887	87	7	68,475,505	rs133520200	0.257	1.00	9.2	− 2.91	0.47	–	Intergenic
30	55	Na	12	73,666,737	73,725,910	47	1	73,674,445	rs136814628	0.680	0.99	8.5	2.53	0.43	HS6ST3	Intron
31	56	K	13	54,405,085	54,637,805	45	8	54,431,192	rs133004626	0.623	0.95	10.2	− 6.48	0.99	–	Intergenic
32	57	Citrate	14	238,942	613,906	35	15	453,437	rs384162250	0.512	1.00	11.0	0.10	0.01	PPP1R16A	Upstream
**32**	**58**	**K**	**14**	**232,311**	**639,312**	**79**	**20**	**515,185**	**rs460225555**	**0.501**	**0.96**	**14.8**	**5.60**	**0.70**	**VPS28**	**Downstream**
32	59	P	14	271,581	758,897	64	20	577,322	rs133788084	0.212	0.21	13.8	12.6	1.64	FBXL6	Upstream
32	60	Ca	14	284,571	700,497	27	7	666,982	rs133196323	0.025	0.34	13.0	32.2	4.31	MROH1	Intron
33	61	Na	15	52,059,631	52,851,538	466	6	52,461,335	rs210668186	0.218	0.60	9.2	− 2.65	0.43	FCHSD2	Intron
34	62	K	17	50,508,807	50,988,913	227	6	50,825,796	rs474259058	0.055	0.95	21.3	15.1	1.56	BRI3BP	Intron
35	63	Ca	18	10,563,963	10,833,324	11	4	10,833,324	rs137549452	0.045	0.91	11.3	− 12.9	1.86	KLHL36 / COTL1	Upstream / Upstream
36	64	Ca	19	56,448,441	56,609,243	340	8	56,524,233	rs207749796	0.335	0.87	16.1	7.39	0.89	FADS6	Intron
**37**	**65**	**Na**	**19**	**60,550,831**	**60,870,548**	**95**	**2**	**60,555,853**	**rs435138644**	**0.783**	**0.61**	**23.6**	**4.40**	**0.43**	**–**	**Intergenic**
37	66	Ca	19	60,009,085	60,870,548	41	1	60,561,566	rs41923848	0.843	0.73	11.2	− 7.19	1.05	–	Intergenic
**38**	**67**	**Ca**	**20**	**31,888,449**	**31,888,449**	**1**	**1**	**31,888,449**	**rs385640152**	**0.009**	**0.91**	**17.0**	**− 25.1**	**2.92**	**GHR / LOC112443004**	**Missense / lncRNA**
39	68	Mg	20	31,888,449	31,888,449	1	2	31,888,449	rs385640152	0.009	0.91	13.9	− 1.85	0.24	GHR / LOC112443004	Missense / lncRNA
39	69	P	20	31,888,449	31,888,449	1	1	31,888,449	rs385640152	0.009	0.91	10.3	− 17.0	2.59	GHR / LOC112443004	Missense / lncRNA
40	75	Ca	20	58,185,895	58,207,145	21	1	58,189,663	rs110048176	0.170	1.00	25.3	13.1	1.24	ENSBTAG00000048498	lncRNA
40	76	Mg	20	58,185,895	58,388,462	65	3	58,189,663	rs110048176	0.170	1.00	117.2	2.34	0.10	ENSBTAG00000048498	lncRNA
**40**	**77**	**Citrate**	**20**	**58,185,895**	**58,388,462**	**81**	**3**	**58,204,929**	**rs109956167**	**0.166**	**0.90**	**187.2**	**0.73**	**0.02**	**ENSBTAG00000048498**	lncRNA
40	78	K	20	58,185,895	58,388,849	69	2	58,386,888	rs134021638	0.054	0.92	26.3	17.4	1.61	ANKH	Intron
**41**	**81**	**Citrate**	**20**	**63,223,010**	**64,759,735**	**87**	**2**	**63,223,010**	**rs109390768**	**0.595**	**0.98**	**14.9**	**0.14**	**0.02**	**–**	**Intergenic**
41	82	Mg	20	63,223,010	64,753,386	13	1	63,223,010	rs109390768	0.595	0.98	9.0	0.47	0.08	–	Intergenic
42	85	Mg	21	39,683,083	41,254,513	144	2	39,933,138	rs134950504	0.070	0.99	9.3	− 0.85	0.14	–	Intergenic
43	86	P	22	28,516,349	30,130,198	35	3	30,130,198	rs108953480	0.966	0.90	8.6	11.4	1.92	FOXP1 / ENSBTAG00000052330	Upstream / lncRNA
**44**	**87**	**P**	**22**	**32,774,174**	**32,787,867**	**10**	**2**	**32,786,684**	**rs208411747**	**0.040**	**0.92**	**20.6**	**− 14.5**	**1.53**	**FAM19A4**	**Intron**
44	88	Mg	22	32,772,562	33,329,180	45	4	32,786,854	rs209198296	0.046	0.82	13.3	− 1.03	0.14	FAM19A4	Intron
45	89	K	24	49,837,381	50,315,415	274	8	50,074,695	rs448650804	0.324	0.61	11.1	5.18	0.76	–	Intergenic
**46**	**90**	**P**	**24**	**58,272,345**	**58,396,158**	**102**	**4**	**58,306,855**	**rs380879212**	**0.362**	**0.56**	**12.8**	**5.26**	**0.71**	**LMAN1**	**Intron**
47	91	Ca	25	20,480,835	20,482,817	2	1	20,480,835	rs109069510	0.888	0.99	8.4	− 8.01	1.36	HS3ST2	Intron
48	92	Mg	27	36,511,563	36,592,556	65	6	36,522,002	rs378026790	0.488	0.96	15.2	0.63	0.08	GPAT4	Upstream
**49**	**93**	**K**	**28**	**6,518,121**	**6,534,988**	**2**	**2**	**6,518,121**	**rs42033936**	**0.224**	**0.98**	**13.0**	**− 6.44**	**0.86**	**KCNK1**	**Intron**
49	94	Na	28	5,987,406	6,889,024	58	3	6,518,121	rs42033936	0.224	0.98	10.8	− 2.70	0.40	KCNK1	Intron
50	95	Ca	29	9,193,304	9,907,275	162	7	9,517,882	rs380735416	0.163	0.41	13.1	8.67	1.16	PICALM	Upstream
**50**	**96**	**Na**	**29**	**8,731,208**	**10,144,903**	**486**	**11**	**9,923,696**	**rs385315120**	**0.166**	**0.87**	**14.8**	**− 4.28**	**0.54**	**TMEM126B**	**Downstream**

**Table 5:** Number of QTL and the percentage of genetic variance explained for each trait.

Trait	Number of QTL	% of genetic variance explained by the QTL		
		Total	Min	Max
Ca	21	33.1	0.43	5.0
P	14	41.7	0.27	23.3
Mg	13	31.0	0.42	10.6
K	14	42.2	0.66	13.6
Na	15	19.8	0.52	2.6
Citrate	6	32.9	0.76	23.1

### Annotation of GWAS peaks

**Figure 3:**
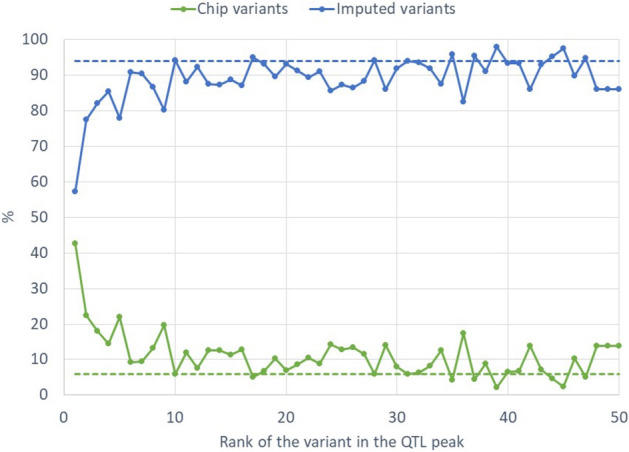
Percentages of chip variants (50 K, HD, or “research” SNPs, in green) and imputed variants (in blue) found among the top 50 variants of QTL peaks. Dashed lines represent the total percentage of chip variants (6%, in green) and imputed variants (94%, in blue) of all variants that were tested in the GWAS.

The sizes of the confidence intervals (CI) of QTL ranged from 1 bp to 3.3 Mbp; each CI contained between 1 and 1004 variants with significant effects (99.3 on average). While only ca. 6% of the tested variants were included on SNP chips, these chip variants (mainly HD) were disproportionately represented among the variants ranked in the top 10 in QTL peaks, and accounted for nearly 43% of the variants ranked first in the peaks, i.e. “top 1” variants (Fig. 3). For less than one-third of the QTL detected (24/83), the variant with the most significant effect was not located in a gene. Depending on the set of significant variants under consideration (all, top 100, top 50, top 10, or top 1), between 25.1% (top 1 variants) and 28.8% (all variants in the CI of the QTL) of variants were intergenic, while 60.5% (top 1) to 65.7% (top 10) of variants were located in genes, including upstream and downstream regions (Table 6). In all QTL, we therefore identified at least one positional candidate gene (1 to 20 per QTL, 5.3 on average). In total, 271 different genes were found within the CIs of the 83 QTL. In the four QTL regions with the most significant effects, the top-ranked variants were located in the genes *SLC37A1* (BTA1), *ANKH* (BTA20), *SEL1L3* (BTA6), and *PPARA* (BTA5); and the top-ranked variants in the remaining QTL were located in dozens of other genes (Tables 3 and 4). The majority of the variants located in genes were intronic, and were only rarely found in coding regions: only 1%, approximately, of variants were non-synonymous, i.e. 84 variants within the CIs of the QTL, and 55, 24, 4, and 1 among the top 100, top 50, top 10 and top 1 variants, respectively (Table 6). Out of these non-synonymous variants located in coding regions of genes, 4 are serious candidates because they were found among the 10 most significant variants of the QTL peaks: G446S in *SLC26A4* (ranked 2nd for the QTL found on BTA4 at 48.7Mbp for Ca), V298M in *ENSBTAG00000050954* (ranked 10th for the QTL found on BTA7 at 40.3Mbp for P), F267V in *ARHGAP39* (ranked 5th for the QTL found on BTA14 at 0.4Mbp for K and Citrate) and F257Y in *GHR* (ranked 1st for the QTL found on BTA20 at 31.9Mbp for P, Ca and Mg). We also found a relatively high proportion of variants located in non-coding RNA (7.9% of all variants in the CIs of the QTL and 13.6% of top 1 variants). As presented in Table 6, these variants were mostly located in long non-coding RNA (lncRNA), less frequently in micro RNA (miRNA) and small nuclear RNA (snRNA), and very rarely in small nucleolar RNA (small Cajal body-specific RNA, scaRNA), and transfer RNA (tRNA).

**Table 6:** Annotation of variants located within confidence intervals of the QTL (a single variant could have different annotations).

Annotation	ALL		Top 100		Top 50		Top 10		Top 1	
	N	%	N	%	N	%	N	%	N	%
Intergenic	3705	28.8	1785	27.3	1062	26.0	269	25.1	29	25.9
Total protein coding	5575	63.3	2745	61.8	1792	62.9	494	65.7	49	60.5
Intron	3223	36.6	1427	32.1	980	34.4	267	35.5	33	40.7
Downstream	1203	11.7	635	12.1	368	11.1	71	8.0	4	4.2
Upstream	948	9.2	562	10.7	378	11.4	136	15.3	9	9.4
3′ UTR	102	1.0	40	0.76	23	0.69	5	0.56	0	0
Missense	84	0.81	55	1.1	24	0.78	4	0.68	1	1.0
Synonymous	76	0.74	38	0.73	28	0.84	10	1.1	3	3.1
5′ UTR	61	0.59	44	0.84	27	0.81	13	1.5	0	0
Splice region	11	0.11	5	0.10	1	0.03	0	0	0	0
Splice acceptor	1	0.01	0	0	0	0	0	0	0	0
Frameshift	1	0.01	0	0.00	0	0	0	0	0	0
Total non-coding RNA	693	7.9	487	11.0	315	11.1	69	9.2	11	13.6
lncRNA	552	8.8	367	11.4	237	11.2	68	12.1	11	18.3
miRNA	69	1.1	50	1.5	42	2.0	1	0.18	0	0.00
scaRNA	4	0.06	4	0.12	4	0.19	0	0.00	0	0.00
snRNA	67	1.1	65	2.0	31	1.5	0	0.00	0	0.00
tRNA	1	0.02	1	0.03	1	0.05	0	0.00	0	0.00
Total	10,311	100	5234	100	3327	100	888	100	96	100

We then completed annotation of the QTL (1) identifying putative regulatory SNPs (rSNPs) located within transcription factor binding sites (TFBSs) according to the procedure described in the Methods section, and (2) using transcriptomic data available on the Cattle Gene Atlas website, http://cattlegeneatlas.roslin.ed.ac.uk/.

In the CIs of 40 of the 83 QTL, we identified 184 variants that overlapped with the TFBSs of 438 transcription factors (TFs), i.e. putative rSNPs (see Supplementary Table S1). These 438 TFs had 67 unique target genes among the 271 positional candidates identified in the QTL. Each TF targeted from 1 to 5 positional candidate genes, and 110 TFs had binding sites (BSs) at multiple loci. One TF (ASCL2) targeted 5 genes (*BHLHE23*, *DGKQ*, *RMND5B*, *bta-let-7a-3*, and *bta-mir-2443*), while 2, 21, and 85 TFs targeted 4, 3, and 2 genes, respectively. Two top 1 variants, rs108972810 (~ 45.4 Mbp in the upstream region of *SMIM20* on BTA6, for Na) and rs209051255 (~ 41.3 Mbp in the upstream region of *ENSBTAG00000053872* on BTA7, for Ca) were in the TFBSs for the TFs DOF and FOXD3, respectively. In total, for 22 QTL, top 10 variants were found in the TFBSs of 19 different target genes (including *CSN2*, *PAEP*, *GPAT4*, *BRI3BP*, and *PPARA*), which are potentially regulated by 47 TFs.

**Figure 4:**
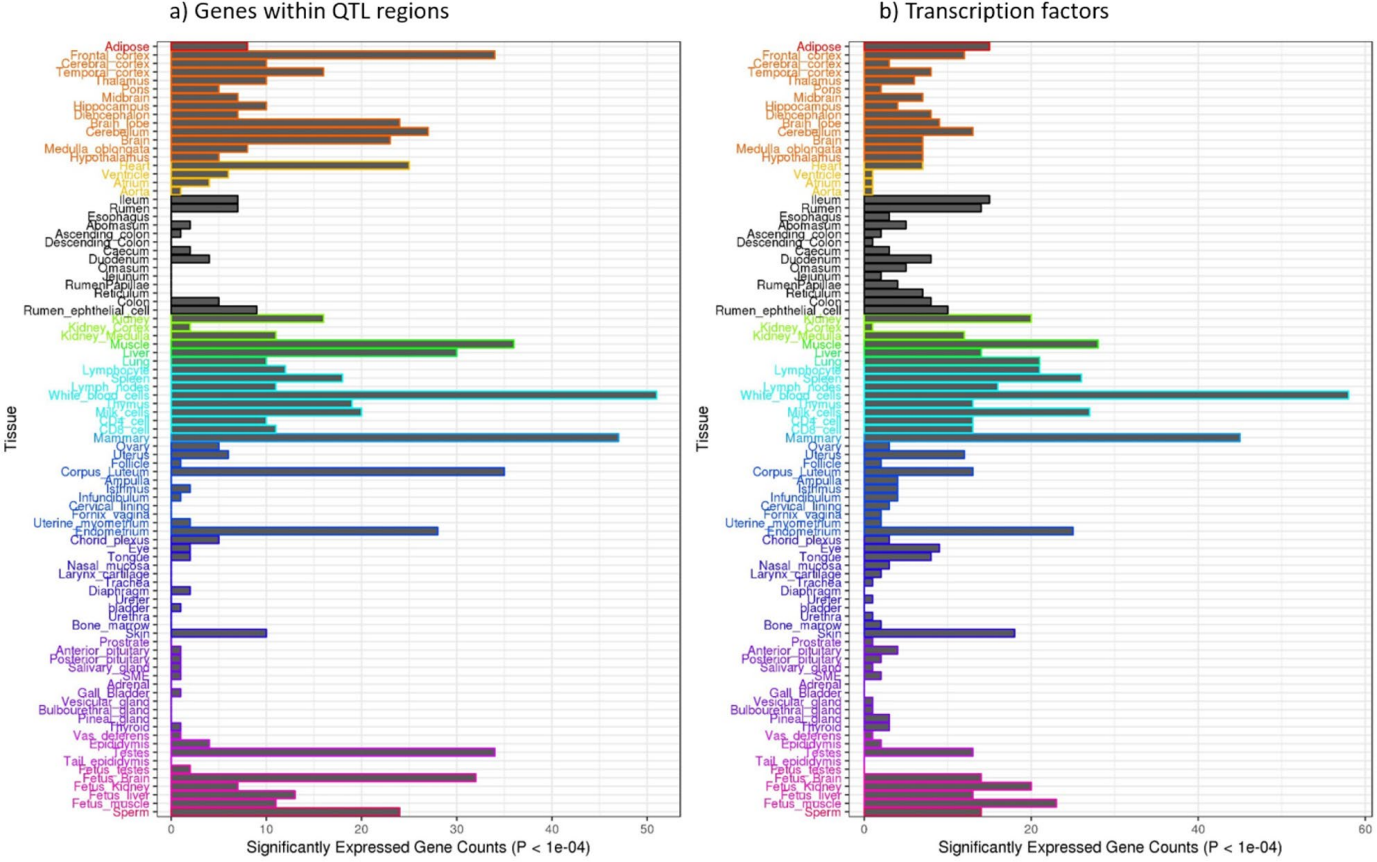
Counts of significantly expressed genes (*P* < 10^−4^) in 91 tissues, showing (**a**) positional candidate genes and (**b**) transcription factors identified as putative regulators of these genes.

We then assessed the degree to which these positional candidate genes and TFs demonstrated tissue-specific expression. Of the 271 genes and 438 TFs associated with milk-mineral QTL, 203 and 160, respectively, were present in the Cattle Gene Atlas, which contains expression data from 91 different tissues or cell lines. Using the model described in the Methods section, we assessed the overexpression or tissue specificity of these genes by evaluating the *t*-statistic and the *P* value, *Pt*, associated with the effect of the tissue under consideration (see Supplementary Tables S2 and S3). Among the 203 positional candidate genes and 160 TFs with a putative regulatory effect on these genes, we calculated the number that were overexpressed in each tissue type (*Pt* < 10^−4^). The profiles obtained for both categories of genes, presented in Fig. 4, were quite similar. The tissues or cell types in which we found the highest number of genes associated with milk mineral composition were white blood cells (51 candidate genes and 59 TFs) and mammary gland (47 candidate genes and 45 TFs). In each tissue, we identified the most-specific genes by retaining the top 10% with respect to *t*-statistic values, i.e. 20 candidate genes and 16 TFs. Among the candidate genes that were most specific to mammary gland, we found the genes encoding the main milk proteins (*CSN1S1*, *CSN1S2*, *CSN2*, *CSN3*, and *PAEP*), and *SLC37A1* and *ANKH*, which are located within the QTL that had the most significant effects in the present study. The ASCL2 TF, which potentially regulates five genes located in the CIs of the QTL, was one of the most mammary-gland-specific TFs. Among genes specific to white blood cells, we found *COTL1*, *MKL1*, and *FOXP1*. *SLC37A1* was not among the 20 most-specific genes but it was ranked 22nd. In the upstream or intronic regions of these four genes, we identified the top-ranked variant of four different QTL, located on BTA18, BTA5, BTA22, and BTA1, respectively. Furthermore, we identified 27 rSNPs for which both TF and target gene were highly specific to the mammary gland; these combinations were composed of 26 unique TFs and 15 unique target genes (see Supplementary Table S4).

### Focus on the *SLC37A1* and *ANKH* gene regions

As mentioned above, two QTL—located on BTA1 (142.8 Mbp) and BTA20 (58.2 Mbp) in the vicinity of the *SLC37A1* and *ANKH* genes, respectively—had very strong effects on milk mineral and citrate content. The *SLC37A1* region affected P, K, Mg, and Na levels while the *ANKH* region had effects on citrate, Mg, K, and Ca (Fig. 2). These two regions alone affected all six milk components analyzed in this study. They explained a high proportion of the genetic variance in P (23.3%), citrate (23.1%), Mg (17.9%, i.e. 7.3% for *SLC37A1* and 10.6% for *ANKH*), and K (15.4%, i.e. 13.6% for *SLC37A1* and 1.8% for *ANKH*), but had much more moderate effects on Na (2.6%) and Ca (2.2%).

In the *SLC37A1* region, the variant with the most significant effect was located in an intron; it was an imputed variant (R^2^ = 0.85) for P and Mg (rs109459130 at 142,834,737 bp) and a chip variant (HD SNP) for K and Na (rs109717634 at 142,835,551 bp). For both variants, which were located 814 bp apart, the most frequent allele (0.58 and 0.61, respectively) increased the amount of all minerals affected by this region.

In the *ANKH* region, we identified three different top 1 variants, depending on the trait. For K, the top variant was rs134021638 (at 58,386,888 bp, imputed with R^2^ = 0.92), located in an intronic region of *ANKH*; for Ca, Mg and citrate, the top variants were located in an lncRNA *ENSBTAG00000048498* (rs110048176 at 58,189,663 bp, HD SNP for Ca and Mg and rs109956167 at 58,204,929 bp, imputed variant with R^2^ = 0.90 for citrate). *ENSBTAG00000048498* spans from 58,186,301 to 58,224,434 bp and is located around 83 kbp upstream of the *ANKH* gene (58,307,527–58,477,497 bp). In all cases, the alleles responsible for an increase in citrate and mineral content were not the most common, with a MAF of 0.05 for the *ANKH* variant (rs134021638) and 0.17 for the two other variants.

**Figure 5:**
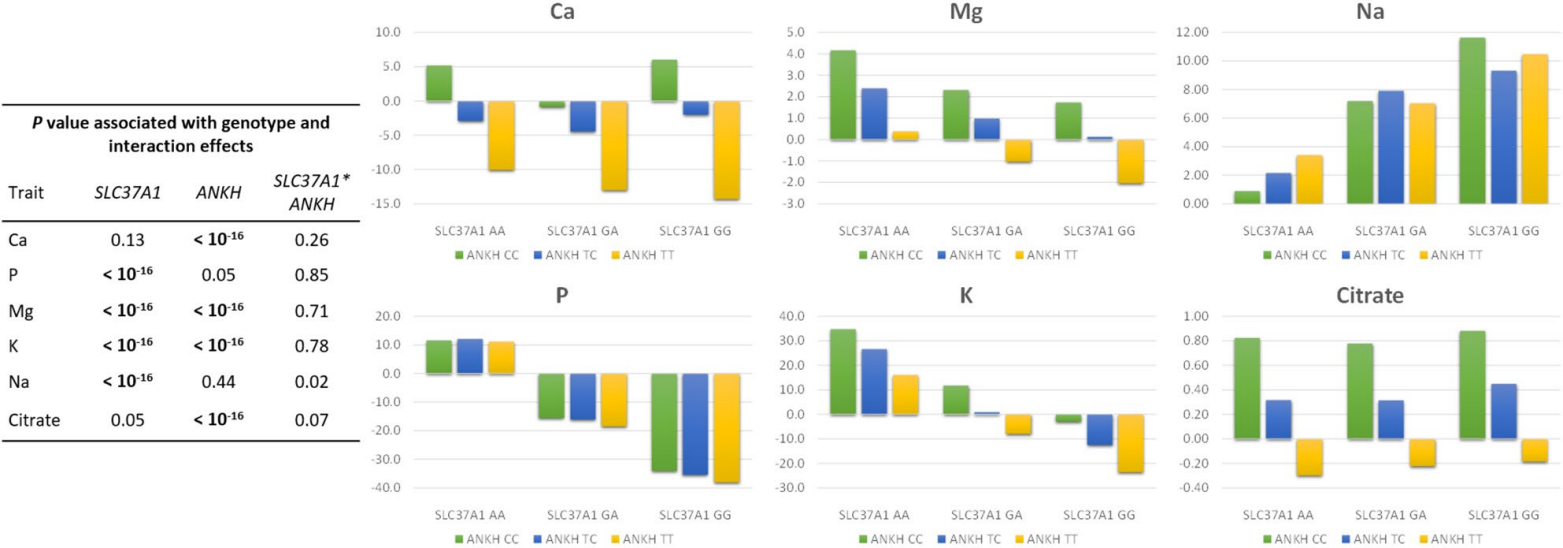
*SLC37A1* and *ANKH* genotypes and genotype interaction effects on milk mineral (Ca, P, Mg, K, and Na) or citrate content.

In both regions, we examined the top-ranked variant for the most-affected traits, i.e. rs109459130 and rs109956167, and tested the interaction effects of their genotypes on milk mineral and citrate content. The results, presented in Fig. 5, revealed no significant interaction for any of the traits analyzed (*P* ≥ 0.02), suggesting that the effects of the two regions on milk composition were additive.

## Discussion

To the best of our knowledge, this study is unique because it is the first GWAS of imputed whole-genome sequences based on the most-recent bovine reference genome with such a large population of cattle (19,586 cows); and it is the first attempt to investigate milk mineral content with a sequence-based GWAS, which here assessed a very large panel of genomic polymorphisms (12.9 million).

Using this approach, we identified 83 QTL that explained a substantial part of the genetic variance (up to 42.2%) for mineral and citrate content in cows’ milk; in each QTL region, we then identified functional candidate genes and variants. Our results build on those of two previous studies that used GWAS to investigate the mineral composition of milk—based on mineral content measured with reference methods and HD 777 k SNP genotypes—in small populations of Holstein (371^9^ and 444^12^) or Jersey (321^9^) cows. The study of Buitenhuis et al.^9^ was dedicated to mineral composition while Kemper et al.^12^ attempted to identify QTL that overlapped between milk production and composition traits. Only two QTL were shared between the two studies: a region located on BTA1 that affected P, for which both studies identified *SLC37A1* as the best functional candidate gene, and a region located near the *DGAT1* gene on BTA14 that affected Ca and P. In our study, we also identified these two regions, together with dozens of other regions located throughout the bovine genome.

By considering a very large dataset (more than 1 million test-day records of 126,873 cows), we estimated heritability values for mineral and citrate composition to be moderate to high, i.e. higher^5,7,8,10^ than or similar^6,9^ to those previously reported in the literature. Furthermore, we found strong genetic correlations among Ca, P, and Mg and among Ca, Mg, and citrate, which was consistent with the studies of Toffanin et al.^5^ and Denholm et al.^8^. Among minerals, Na had both the lowest heritability value (0.32 vs 0.48–0.56 for the other minerals) and the smallest percentage of genetic variance explained by its associated QTL (19.8% vs 33.1–42.2% for the other minerals). Ca, P, and Mg shared more QTL than any other group of minerals: 7, 6, and 6 QTL were shared between Ca and P, Ca and Mg, and P and Mg, respectively, while 4 QTL had effects on all three. In contrast, despite the high degree of genetic correlation between Ca, Mg, and citrate, only 2 QTL were shared among these three traits; however, one of these was a QTL located on BTA20 (~ 58.2 Mbp) that explained 2.2%, 10.6%, and 23.1% of the genetic variance of Ca, Mg, and citrate, respectively. Overall, estimates of genetic correlations were consistent with QTL results, which probably reflects the common biological pathway of these minerals in milk. In colloidal form, Ca, P, and Mg are associated with caseins in the micelles while in the soluble fraction, Ca and Mg are associated with citrate. Further studies, using random regression models fitted across lactation, could investigate the pattern of genetic relationships between the different milk minerals during lactation and thus provide a better understanding of the underlying biological mechanisms.

The resolution of our study was high enough to identify a single or a few positional candidate genes in most of the 83 QTL we identified. In each of these regions, though, the variant with the most significant effect was not necessarily located in a gene. As an example, we detected the overrepresentation of chip SNPs at the top of the QTL peaks (42.7% of the top 1 variants), and the majority of these SNPs were located in intergenic regions. Chip SNPs are directly genotyped or have a higher imputation accuracy than the surrounding variants, which probably enabled more precise estimation of their effects. To identify the best candidate variant(s) for each QTL, we therefore considered not only the variant with the most significant effect, but the set of variants ranked at the top of the peak that were located in or close to genes. In most cases, we found that these variants were located in non-coding regions of genes; for example, 15.3% and 12.1% of all top 10 variants were located in upstream regions of genes or in lncRNA, respectively, i.e. in putative regulatory regions. To further refine our results, we consulted in silico annotations of rSNPs in upstream regions of genes as well as existing knowledge regarding genomic regions that are transcribed into non-coding RNA. Moreover, to support the putative roles of these variants, we also considered the tissue specificity of candidate genes and transcription factors. Unfortunately, the expression dataset, which was based on Ensembl release 94, did not contain all of the genes located in QTL regions. Here we present some examples of QTL in which the putative causal mutation was located in a regulatory region, with particular attention to functional candidate genes previously associated with milk composition and the QTL with the most significant effects in this study.

*GPAT4* (*glycerol-3-phosphate acyltransferase 4*) was the best candidate gene for the QTL on BTA 27 with effects on Mg. Although it was not the most notable QTL in terms of its effect on minerals, this region, and this gene in particular, have been highlighted by previous studies as affecting milk composition (fat, protein, and lactose content)^19,22–24^. In these studies, five variants in linkage disequilibrium—four in the upstream region and one in the 5′ UTR region of *GPAT4*—were highlighted as the best candidate causal variants. In our study, these variants were ranked in the top 5 in the GWAS peak; the variants ranked 3rd (rs209479876) and 5th (rs209855549) alter the WRKY48 and TWI transcription factor binding sites (TFBSs), respectively, and are more likely to be the causal variants. Daetwyler et al.^23^ also highlighted rs209479876 as the best causal variant in this region because of the high probability that it overlaps a TFBS. We also confirmed that *GPAT4* was overexpressed in the mammary glands (*P* = 3.10^−12^) compared to 90 other tissues or cell types, but expression data were not available for the TFs associated with this gene.

For the QTL identified on BTA11 at ~ 103.2 Mbp with effects on P and citrate, the top two variants in the peak for P (the most-affected trait) were intergenic. Instead, the variants ranked 3rd to 7th were located in the upstream region of the *PAEP* (*progestogen-associated endometrial protein*) gene; the 5th-ranked variant, rs110710904, probably alters a binding site of the Macho-1 transcription factor (*P* = 0.02). *PAEP*, which is one of the most mammary-gland-specific genes (*Pt* = 1.8.10^−37^), encodes β-lactoglobulin, the most abundant whey protein in cow milk. Two non-synonymous variants in this gene, rs109625649 and rs110066229, were previously highlighted as the causal mutations underlying β-lactoglobulin concentration in milk^25^. In our study, these were respectively ranked 157th and 171st in the peak, i.e. far below the top-ranked variants upstream. These results corroborate previous reports that these two putatively causative missense mutations did not explain all the effects of the region on milk composition^19,20^ and highlight an rSNP as a likely causative variant.

*BRI3BP* (*BRI3 binding protein*), located on BTA 17 at ~ 50.8 Mbp, has been previously associated with de novo short chain fatty acid synthesis in bovine milk^26,27^. In our study, this region appeared to have effects on the K content of milk, and we found that *BRI3BP* was significantly overexpressed in mammary gland compared to the 90 other tissues investigated (*P* = 3.9.10^−8^). The first seven variants in the peak were located in an intronic region of *BRI3BP*. The 8th (rs477456528) and 9th (rs440703666) variants, instead, were located in the upstream region of the gene and rs440703666 probably alter the binding site of the transcription factor SPL8. This variant thus represents a very attractive functional candidate in this region.

In the four regions with the most significant effects on milk minerals, we identified *SLC37A1* (BTA 1), *ANKH* (BTA 20), *SEL1L3* / *SMIM20* (BTA 6), and *PPARA* (BTA 5) as the best candidate genes.

Five of the six traits analyzed in this study were affected by a QTL region at ~ 116.4 Mbp on BTA5. Here, a variant located at 116,438,773 bp (1000G_80994138) ranked 2nd for four traits. This variant was located in the upstream region of *PPARA* (*peroxisome proliferator activated receptor alpha*) and overlapped the binding sites for the transcription factors E2F4, RSC3, RSC30, TDA9, and TFDP1. In this QTL region, top 1 variants were located either in an intronic region of *PPARA* (citrate, Mg, and P) or in an lncRNA, *LOC101903383* (Ca and Na). *PPARA* encodes the PPAR-α transcription factor, a key regulator of lipid metabolism belonging to the superfamily of *PPAR* hormone receptors^28^; expression of this TF in mammary gland was previously found to be associated with milk fatty-acid composition in dairy cows^29,30^. Here, we did not detect any mammary-gland specificity of *PPARA* but we did observe that this gene was expressed in this tissue. It is therefore possible that polymorphisms in the binding sites of this TF or in the lncRNA *LOC101903383*, located 1.3 kbp upstream of *PPARA*, could regulate the expression of *PPARA* and be responsible for the strong effects of this region on milk composition.

On BTA6 at ~ 45.3 Mbp, we identified a QTL with very strong significant effects on K and to a lesser extent on P, Na, and Ca. Within the confidence interval of this QTL, we found 80 different potential variants, of which the majority (69) were intergenic, 9 were located in an intronic region of *SEL1L3* (*SEL1L family member 3*), and 2 were in the upstream region of *SMIM20* (*small integral membrane protein 20*). However, when we examined only the top 10 variants for the different traits, the number of distinct variants shrank to 18 (9 intergenic, 7 in *SEL1L3*, and 2 in *SMIM20*). Of these, two (rs108972810 at 45,401,485 bp and rs136498639 at 45,401,570 bp) were located in the upstream region of *SMIM20* and overlapped a TFBS. *SEL1L3* was previously identified as a candidate gene in a QTL region with effects on bovine milk protein composition^19^. Instead, to the best of our knowledge, this study is the first to propose *SMIM20* as a candidate gene for milk composition. However, the functional link between these genes and milk composition has yet to be established, as we found both genes to be endometrium-specific and underexpressed in mammary glands compared to the 90 other tissues investigated.

For milk mineral composition, the two best functional candidate genes highlighted by our analysis were *SLC37A1* (*solute carrier family 37, member A1*) and *ANKH* (*inorganic pyrophosphate transport regulator*). These two genes were previously found to be overexpressed in mammary glands relative to 17 other types of tissue^31^, and here we found both among the top 10% of mammary-gland-specific genes (*Pt* = 3.4.10^−26^ and 6.6.10^−16^, respectively). This suggests that the main function of these genes occurs in epithelial cells of this tissue. *SLC37A1* and *ANKH* both encode transmembrane proteins involved in ion transport and have been found to play a role in inorganic anion transport^20^. Variants located in both genes explained a large degree of the genetic variation in milk mineral content. Earlier studies also linked *SLC37A1* with mineral content, in particular that of P^9,12^, while both genes have been implicated in determining milk protein composition in Holstein, Montbéliarde, and Normande cows^19^ and cheese-making traits in the same Montbéliarde cows we analyzed here^20^. These results are consistent with what is known about the association of minerals with casein molecules in micelles; milk mineral composition is strongly related to milk protein composition^2^ and therefore to cheese-making traits^3,4^.

In *SLC37A1*, 7, 31, 9, and 7 variants were located in the confidence intervals of QTL for P, K, Mg, and Na, respectively. These variants (32 of which were unique) were overwhelmingly located in introns of the gene, in a 12.3-kbp-region from 142,826,156 to 142,838,477 bp. Among these 32 variants, we were not able to distinguish the best candidate based on annotation, but two variants, 814 bp apart, were particularly highly ranked for all traits. Specifically, rs109459130, at 142,834,737 bp, was ranked 1st, 2nd, 1st, and 3rd for P, K, Mg, and Na, respectively, while rs109717634, at 142,835,551 bp, was ranked 2nd, 1st, 3rd, and 1st for P, K, Mg, and Na, respectively. Of these, the first (rs109459130) appeared to be the better candidate because it was top-ranked for the most affected trait, P, and because it was imputed (R^2^ = 0.85), while the second variant was an HD SNP (R^2^ = 0.997).

In the region of the *ANKH* gene, we found 81, 65, 69, and 21 variants in the confidence intervals of QTL for citrate, Mg, K, and Ca, respectively. These represented 82 unique variants, all located either in intronic regions of the *ANKH* gene (59 variants between 58,344,839 and 58,388,849 bp) or in the lncRNA *ENSBTAG00000048498* (23 variants between 58,185,895 and 58,212,187 bp). The same variant was ranked first for both Mg and Ca. Two top-1 variants were located in the lncRNA *ENSBTAG00000048498—*rs109956167 (at 58,204,929 bp) for citrate and rs110048176 (at 58,189,663 bp) for Mg and Ca—while the top-ranked variant for K was located in *ANKH* (rs134021638 at 58,386,888 bp). We propose rs109956167 as the best candidate causal variant because i) it was ranked 1st for the most-affected trait (citrate), ii) it was imputed, in contrast to rs110048176 which was from the HD chip, and iii) it was the only variant present in the top 10 for all traits affected by this region (4th for Mg, 6th for Ca, and 9th for K). We hypothesize that the lncRNA *ENSBTAG00000048498*, which is 83 kbp downstream of the closest gene, *ANKH*, may affect milk mineral content by regulating the expression of *ANKH* and thus the amount of protein available for ion transport.

When we examined the best candidate variants in the regions of *SLC37A1* (rs109459130) and *ANKH* (rs109956167), we found that alleles of these genes did not have significant interactions with respect to the six milk composition traits analyzed in this study and that, combined together, these two regions explained a large part of the genetic variance in milk mineral content. The allele that increased mineral content was the most frequent allele of *SLC37A1* (allele *A*, frequency = 0.58), while it was the least frequent allele in the *ANKH* region (allele *C*, frequency = 0.17).

Compared to previous studies, our GWAS-based investigation of milk mineral composition was conducted at the whole-genome level, after imputation with the most recent reference genome and using data from a large population of animals. This approach led to the identification of a large number of genomic regions that explain a great deal of the genetic variance of the traits studied here. Moreover, post-GWAS investigations conducted using different annotation datasets enabled the identification of candidate causal genes and the prioritization of candidate variants in these genomic regions. In most situations, exonic variants were not proposed as the best candidates, although they cannot be excluded in four QTL regions, including *GHR*. In contrast, the best candidate variants often were putative regulatory variants. Although the functional causative effect of the candidate variants remains to be demonstrated, this study highlights a ‘short list’ of candidate variants, in particular rs109459130 (*SLC37A1*) and rs109956167 (*ANKH*), whose favorable alleles can be feasibly selected to increase the mineral content (Ca, P, Mg, and K) of milk and therefore improve both its nutritional and cheese-making qualities in Montbéliarde cows.

## Methods

### Ethics statement

Milk samples were analyzed with MIR spectrometry during routine milk recording in commercial herds of Montbéliarde cows in the Franche-Comté region (France). We did not perform any experiments on animals and no ethical approval was required.

### Animals and phenotypes

The original dataset was generated by the From’MIR project and is described in detail by Sanchez et al.^11,20^. It comprised 6,670,769 mid-infrared (MIR) spectra of milk samples from 410,622 Montbéliarde cows. The concentrations of five milk minerals (Ca, P, Mg, K, and Na) and citrate were predicted from MIR spectra using equations developed by the Optimir project^14,15^ (Table 1). As previously described^11,20^, to ensure that the dataset was homogeneous (and less computationally demanding), we retained only the first-lactation records with at least seven test-day records per cow for estimating variance components (1,100,238 test-day records from 126,873 cows^11^). To estimate environmental effects used to derive the phenotypes included in GWAS, at least three test-day records per cow were required, corresponding to a dataset of 1,442,371 test-day records from 189,817 cows^20^.

### Estimation of genetic parameters

First lactation data were analyzed using bivariate repeatability animal models applied to all pairs of traits. (Co)variance components were estimated using the AI-REML algorithm as implemented in Wombat software^32^, with the following linear animal model:

1 $$ {\mathbf{y}} = {\mathbf{X\beta }} + {\mathbf{Za}} + {\mathbf{Wp}} + {\mathbf{e}}_{1} , $$

where ${\mathbf{y}}$ is the vector of test-day observations; **a ~ **${\mathbf{N}}\left( {0,{\mathbf{A}} \otimes {\mathbf{G}}_{0} } \right)$ is the vector of random additive genetic effects, **p ~ **${\mathbf{N}}\left( {0,{\mathbf{I}} \otimes {\mathbf{P}}_{0} } \right)$ is the vector of random permanent environmental effects, and ${\varvec{e}}_{1}$ ~ ${\mathbf{N}}\left( {0,{\mathbf{I}} \otimes {\mathbf{R}}_{0} } \right)$ is the vector of random residual effects.**X, Z,** and **W** are incidence matrices, **A** is the relationship matrix among individuals calculated from the pedigree (traced back over four generations and containing 315,661 animals), and **I** the identity matrix. ${\mathbf{G}}_{0}$**,** ${\mathbf{P}}_{0}$ and ${\mathbf{R}}_{0}$ are 2 × 2 matrices of additive genetic, permanent environmental, and residual variances-covariances, respectively. The ${{\varvec{\upbeta}}}$ vector included the fixed effects of the herd x test-day x spectrometer combination, stage of lactation, and season of calving.

### Genotypes and imputation to whole-genome sequences

A subset of 19,586 cows for which MIR spectra were available had been genotyped for the purpose of genomic selection using the BovineSNP50 (50 K, 6505 cows) or the EuroG10K BeadChip (Illumina Inc., San Diego, 13,081 cows); the latter contains generic SNPs and a research add-on for causal or predictive SNPs for traits of interest in cattle. Missing genotypes were imputed using FImpute software^33^ for the 53,469 autosomal SNPs (50 K and “research” SNPs, 50 K+) that passed all quality control filters (individual call rate > 95%; SNP call rate > 90%; minor allele frequency (MAF) > 1% in at least one major French dairy cattle breed; genotype frequencies in HW equilibrium with *P* > 10^−4^). Whole-genome sequences (WGSs) were then imputed in two steps. In the first step, 777 K high-density and “research” SNPs (HD+) were imputed with FImpute software using a within-breed reference set of 522 Montbéliarde bulls genotyped with the Illumina BovineHD BeadChip (Illumina Inc., San Diego, CA)^34^. Finally, allele dosages of the WGSs were imputed using Minimac software^35^ and WGS variants of 1479 *Bos taurus* animals from the 7^th^ run of the 1000 Bull Genomes Project, representing 17 cattle breeds and including 63 Montbéliarde bulls. This 2-step strategy was found to be the most accurate^36^ (eg, Boowman & Veerkamp, 2014) because (1) the first step takes advantage of the high number of HD genotypes of influential bulls and is performed with very limited loss in accuracy^34^ (Hozé et al., 2013) ; (2) the number of sequenced Montbéliarde bulls was too limited for an accurate imputation to sequence when used alone; and (3) linkage disequilibrium is partially conserved across breeds at the HD level (~ several kb), making use of close breeds sequence data very beneficial. WGS variants were selected following the protocol defined by the 1000 Bull Genomes consortium^16,23^, as described in Boussaha et al.^37^. Short reads were filtered for quality and aligned to the ARS-UCD1.2 reference sequence^17^, and small genomic variations (SNPs and InDels) were detected using SAMtools 0.0.18^38^. Raw variants were then filtered to produce a dataset of 25,050,323 variants. The precision of imputation from HD+ to WGS was assessed using R^2^ values calculated with Minimac software^35^. Only variants with R^2^ ≥ 0.20 and MAF ≥ 0.005 were retained for association analyses, i.e. 12,907,802 variants, with a mean R^2^ of 0.67 and MAF of 0.19.

### GWAS

We performed single-trait association analyses between all 12,907,802 polymorphic variants and each of six milk mineral and citrate traits, described in Table 1. All association analyses were performed using the *mlma* option of GCTA software (version 1.24), which applies a mixed linear model that includes the variant to be tested^39^:

2 $$ {\mathbf{yd}}{ } ={\mathbf{1}}m + { }{\mathbf{x}}_{{\mathbf{v}}} {\text{b}}_{{\text{v}}} + { }{\mathbf{u}}{ } + { }{{\varvec{\upvarepsilon}}}, $$

where ${\mathbf{yd}}$ is the vector of so-called yield deviations, i.e. test-day records adjusted for non-genetic effects with the mixed linear model (1) using Genekit software^40^ and averaged per cow; $m$ is the overall mean; ${\text{b}}_{{\text{v}}}$ is the additive fixed effect of the variant to be tested for association; ${\mathbf{x}}_{{\mathbf{v}}}$ is the vector of imputed allele dosages, ranging from 0 to 2; ${\varvec{u}} \sim N\left( {0, \user2{G\sigma }_{u}^{2} } \right)$ is the vector of random polygenic effects, with ${\varvec{G}}$ the genomic relationship matrix (GRM) calculated using the HD SNP genotypes (which offer both high density and accuracy of imputation), and $\sigma_{u}^{2}$ the polygenic variance, estimated based on the null model $\left( {{\mathbf{yd}} = {\mathbf{1}}m + {\mathbf{u}} + {\mathbf{e}}} \right)$ and then assumed as known while testing for the association between each variant and the trait of interest; and ${\varvec{\varepsilon}} \sim N\left( {0, I{\varvec{\sigma}}_{{\varvec{\varepsilon}}}^{2} } \right)$ is the vector of random residual effects, with ***I*** the identity matrix and $\sigma^{2}$ the residual variance. Association was tested using a *t*-statistic calculated by dividing the variant effect estimate by its standard error.

In order to correct for multiple testing, the Bonferroni correction was applied to take into account all 12.9 million independent tests. The 5% genome-wide threshold of significance therefore corresponded to a nominal *P*-value of 4 × 10^−9^ (-log_10_(*P*) = 8.4) per test. When a given trait was significantly affected by multiple variants, variants that were located less than 2 million base-pairs (Mbp) apart were grouped together to define QTL, considering the variants belonged to the same QTL region. The bounds of the confidence intervals (CIs) of each region were then determined based on the positions of variants that were included in the upper third of the QTL peak.

In regions where multiple neighboring QTL were identified (BTA1 and BTA20), conditional analyses were carried out using the *cojo* option of GCTA^39^ in order to conclude if multiple significant variants in a genomic region were due to LD with the same causal mutation or to the presence of multiple causal mutations. Association analyses were performed by including in the model the most significant variant as a fixed effect and by testing all variants in these neighboring QTL that were not in strong LD with the conditional variant (r^2^ < 0.9).

### Functional annotations

Genomic regions and variants were annotated with FAANGMine v1.1 (https://faangmine.elsiklab.missouri.edu/), developed by the Functional Annotation of ANimal Genomes initiative^18^ and which integrates the ARS-UCD1.2 bovine reference genome with a variety of external data sources, including RefSeq from NCBI (https://www.ncbi.nlm.nih.gov) and Ensembl (https://www.ensembl.org) gene sets.

The ability of genetic variants to alter transcription factor binding sites (TFBSs) was predicted with a custom script that used TFBS models from the JASPAR (JASPAR CORE 2018 collection^41^), HOCOMOCO (version v10^42^), and TRANSFAC (version v3.2 public^43^) databases. These databases contain curated sets of transcription factor binding models represented as Position Weight Matrices (PWM), which are derived from published collections of experimentally defined eukaryote TFBSs. Only vertebrate PWMs were downloaded for use in our study.

Gene overexpression or specificity in different tissues was determined using gene expression patterns of 24,616 genes (Ensembl release 94) available in the Cattle Gene Atlas, which contains 723 RNA-seq datasets representing 91 tissues and cell types, classified into 17 biological categories (http://cattlegeneatlas.roslin.ed.ac.uk/). To assess the expression specificity of each gene in a given type of tissue (by excluding tissues in the same biological category), we applied the following linear model as described in Fang et al.^44^:

3 $$ {\mathbf{ye}}{ } = {\mathbf{1}}me + { }{\mathbf{x}}_{{\mathbf{t}}} {\text{b}}_{{\text{t}}} + { }{\mathbf{zc}}{ } + { }{\mathbf{ee}} $$

where ${\mathbf{ye}}$ is the vector of expression level in the tissues, assessed by the scaled log_2_FPKM (Fragments Per Kilobase per Million mapped reads); $me$ is the overall mean; ${\mathbf{x}}_{{\mathbf{t}}}$ is the vector of the variable with value 1 for samples of the tested tissue and − 1 for samples outside the same category; ${\text{b}}_{{\text{t}}}$ is the corresponding tissue effect; ${\mathbf{z}}$ is the incidence matrix related to the corresponding covariables effects ${\mathbf{c}}$, including age, sex and study effects; ${\mathbf{ee}}$ is the residual effect. Model (3) was implemented adapting R scripts available on the Cattle Gene Atlas website. For each gene, a *t*-statistic was computed by dividing the tissue effect by its standard error. A gene was considered to be overexpressed in a tissue if the probability associated with the *t*-statistic (*Pt*) was lower than 10^−4^. To determine the tissue specificity of genes, we ranked the genes in each type of tissue by their *t*-statistics; the top 10% were considered to be tissue-specific.

### *SLC37A1*-*ANKH* genotype interactions

We tested putative interaction effects between the genes *SLC37A1* and *ANKH* on milk mineral and citrate content with the following mixed linear model:

4 $$ {\mathbf{yd}} = {\mathbf{1}}mi + { }{\mathbf{Mg}}_{{{\text{SLC}}37{\text{A}}1}} { } + { }{\mathbf{Ng}}_{{{\text{ANKH}}}} + {\mathbf{Og}}_{{{\text{SLC}}37{\text{A}}1}} {\text{ x }}{\mathbf{g}}_{{{\text{ANKH}}}} + { }{\mathbf{Ps}}{ } + { }{\mathbf{ei}}, $$

where ${\mathbf{yd}}$ as defined in (2); ${\text{mi}}$ is the overall mean; ${\text{g}}_{{{\text{SLC}}37{\text{A}}1}}$ and ${\text{g}}_{{{\text{ANKH}}}}$ are the fixed effects of the genotypes of the best candidate variant in the *SLC37A1* and *ANKH* regions, respectively; ${\text{g}}_{{{\text{SLC}}37{\text{A}}1}} {\text{ x g}}_{{{\text{ANKH}}}}$ represents the interaction between the two genotypes;$\user2{ }{\mathbf{M}}, {\mathbf{N}}$, and ${\mathbf{O}}$ are incidence matrices related to the individual effects of the *SLC37A1* and *ANKH* genotypes and their interaction, respectively; ${\mathbf{s}}$ is the vector of random sire effects and **P** the corresponding incidence matrix; and ${\mathbf{ei}}$ is the vector of random residual effects. All effects were tested using *t*-statistics computed in the MIXED procedure of SAS software.

## Supplementary Information

Supplementary Information

## Data Availability

The data (genotypes and phenotypes) that enabled the findings of this study were made available by UMOTEST, CEL25-90, and GENIATEST. However, restrictions apply to the availability of these commercial data: they were used under license for the current study, and are not publicly available.
